# A Brain-Computer Interface Based Attention Training Program for Treating Attention Deficit Hyperactivity Disorder

**DOI:** 10.1371/journal.pone.0046692

**Published:** 2012-10-24

**Authors:** Choon Guan Lim, Tih Shih Lee, Cuntai Guan, Daniel Shuen Sheng Fung, Yudong Zhao, Stephanie Sze Wei Teng, Haihong Zhang, K. Ranga Rama Krishnan

**Affiliations:** 1 Department of Child and Adolescent Psychiatry, Institute of Mental Health, Singapore, Singapore; 2 Duke-National University Singapore Graduate Medical School, Singapore, Singapore; 3 Institute for Infocomm Research, Agency for Science, Technology and Research, Singapore, Singapore; 4 Singapore Clinical Research Institute, Singapore, Singapore; Hangzhou Normal University, China

## Abstract

Attention deficit hyperactivity disorder (ADHD) symptoms can be difficult to treat. We previously reported that a 20-session brain-computer interface (BCI) attention training programme improved ADHD symptoms. Here, we investigated a new more intensive BCI-based attention training game system on 20 unmedicated ADHD children (16 males, 4 females) with significant inattentive symptoms (combined and inattentive ADHD subtypes). This new system monitored attention through a head band with dry EEG sensors, which was used to drive a feed forward game. The system was calibrated for each user by measuring the EEG parameters during a Stroop task. Treatment consisted of an 8-week training comprising 24 sessions followed by 3 once-monthly booster training sessions. Following intervention, both parent-rated inattentive and hyperactive-impulsive symptoms on the ADHD Rating Scale showed significant improvement. At week 8, the mean improvement was −4.6 (5.9) and −4.7 (5.6) respectively for inattentive symptoms and hyperactive-impulsive symptoms (both p<0.01). Cohen’s d effect size for inattentive symptoms was large at 0.78 at week 8 and 0.84 at week 24 (post-boosters). Further analysis showed that the change in the EEG based BCI ADHD severity measure correlated with the change ADHD Rating Scale scores. The BCI-based attention training game system is a potential new treatment for ADHD.

**Trial Registration:**

ClinicalTrials.gov NCT01344044

## Introduction

Attention deficit hyperactivity disorder (ADHD), a childhood onset developmental disorder, is a chronic condition that can extend into adulthood [1], [2]. Standard treatment for ADHD includes mainly medication and psychosocial or behavioral treatment [3]. EEG based biofeedback systems have been developed as an alternative modality for treating ADHD. Neurofeedback therapy was developed based on the knowledge that children with ADHD exhibited specific EEG patterns, and EEG feedback training directed at normalizing these rhythms might yield sustaining clinical benefits [4], [5]. Although these systems have been deployed in patient care settings, evidence to support the efficacy for these systems is currently not strong [6], [7]. The EEG based systems try to train the individual to a particular profile of EEG. This profile is not individualized but based on group dynamics. We developed another approach where the EEG profile for attention in a given individual is used to run a game thereby the individual learns to develop increasing attention while playing a game, the brain computer interface (BCI)-based attention training game system.

We previously reported the results of this approach in a small controlled trial [8]. The BCI-based attention training game system utilized filter banks to cover a broad range of EEG rhythms, together with common spatial pattern filtering to determine user-specific spatial-spectral patterns in the EEG for discriminating attentive and inattentive states. The system then transformed the patterns into a variable which represents attentive state or inattentive state. The system was calibrated for each individual using an attention task, the Colour Stroop test, which has been utilized widely in research to assess for attention and response inhibition [9].

In our first study we found that intervention with a training program involving the BCI-based attention training game system improved parent-reported inattentive symptoms. We also noted that behavioural improvement was sustained 3 months after the intensive 20-session intervention. The initial study was 10 weeks in duration with 2 sessions per week. It also involved the use of a very simple game and a tethered connection to a computer. Since then, we have developed a new version of the device that is simple, uses dry EEG electrodes and is connected by Bluetooth to the computer. A new game that could be calibrated based on the performance of the child was also developed. In addition it was important to understand who would be the right candidates to benefit from this approach.

Therefore, the purpose of this study was to investigate if

1. The new device with a new game and a more intensive new training schedule 3 sessions per week over 8 weeks was acceptable to patients.
2. There is preliminary evidence of efficacy in improving ADHD symptoms.
3. There is any clinical predictor of response.
4. There is any EEG change as a function of response.

## Methods

The protocol for this trial and supporting CONSORT checklist are available as supporting information; see Protocol S1 and Checklist S1.

### Ethics Statement

This study was approved by the ethics review boards of the Institute of Mental Health (Clinical Research Committee) and National Healthcare Group, Singapore (Domain Specific Review Board, Domain A). Written informed consent from parents and assent from children were obtained prior to study entry (Clinicaltrials.gov registration no. NCT01344044).

### Study Design

**Figure 1:**
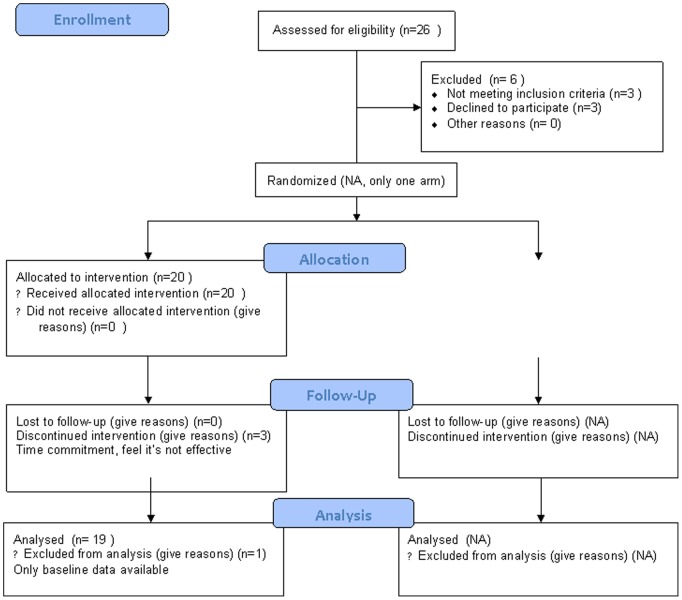
CONSORT flow diagram.

This was a one-arm prospective study and we aimed to enroll 20 children, who would receive treatment with the BCI-based attention training program. The Consort Flow Diagram is shown in Figure 1.

### Participants

We invited the clinic’s psychiatrists to refer patients clinically diagnosed to have the inattentive or combined subtypes of ADHD based on the Diagnostic and Statistical Manual of Mental Disorders-Fourth Edition (DSM-IV) for the study. During the screening phase, parents completed the Diagnostic Interview Schedule for Children Version IV (DISC-IV) [10]. If a potential participant failed a subject in school and/or was enrolled in the school’s Learning Support Program, the Kaufman Brief Intelligence Test, Second Edition (KBIT-II) [11] was administered to assess the intellectual functioning. The inclusion and exclusion criteria for the study were as follows.

#### Inclusion criteria

A subject was eligible for inclusion in the study only if all the following criteria applied at pre-study screening:

- Subject’s age was within the age range of 6–12 years old;
- Subject had never received treatment with stimulant medication or Atomoxetine;
- The subject should satisfy the following criteria for the diagnosis of ADHD:
  1. DSM-IV-TR criteria for ADHD, either the combined or inattentive subtype, based on clinical assessment
  2. Diagnostic Interview Schedule for Children (DISC), as completed by the parents;
- Written Informed Consent from parent and Assent Form from child were both obtained;
- Subject and the parent/guardian were willing to comply with study procedures and were able to return to the clinic for scheduled visits.

#### Exclusion criteria

A subject was not eligible for inclusion in the study if any of the following criteria applied at pre-study screening:

- Present or history of medical treatment with stimulant medication and Atomoxetine;
- Co-morbid severe psychiatric condition or known sensori-neural deficit e.g. complete blindness or deafness (such that they could not play computer games);
- History of epileptic seizures;
- Known mental retardation (i.e. IQ 70 and below);
- Predominantly Hyperactive/impulsive subtype of ADHD (i.e. no predominant inattentive symptoms).

We recruited 20 participants for the study, including 16 males and 4 females. The mean age was 7.80 (SD = 1.40, range 6–11). There were 17 Chinese, 2 Eurasians and one Malay. Fourteen children were diagnosed to have the combined subtype of ADHD based on C-DISC, and the other 6 had the inattentive subtype of ADHD.

### The BCI-based Attention Training Game System

**Figure 2:**
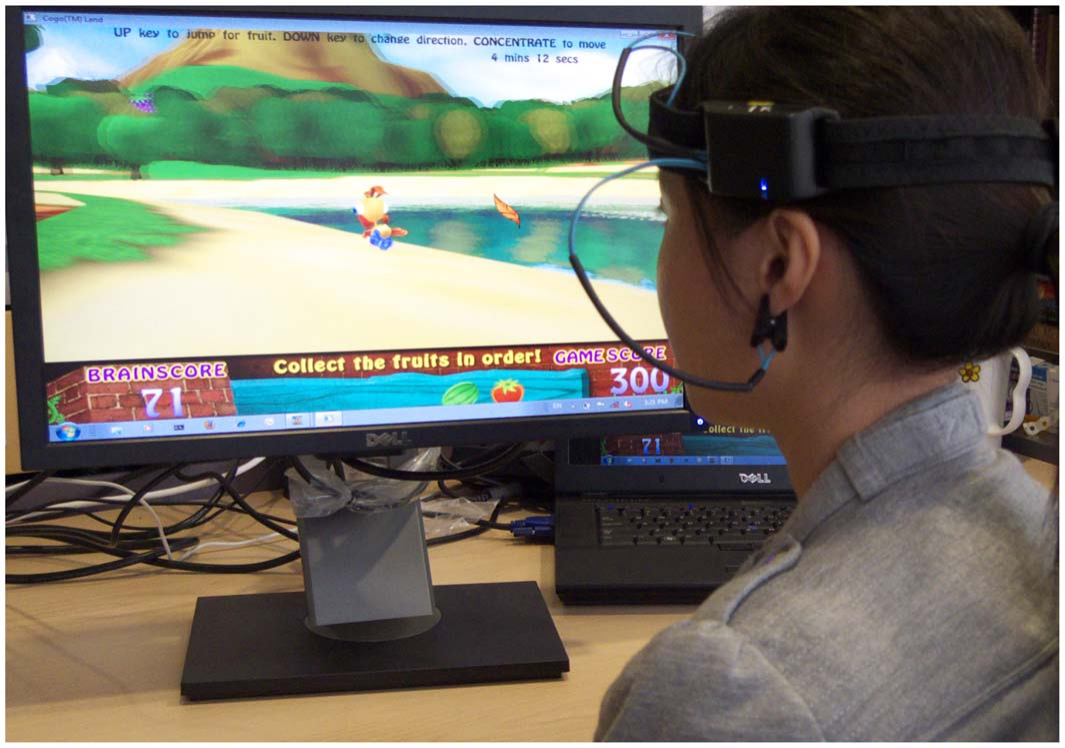
A model engaged in intervention with the Brain-Computer Interface (BCI) attention training game system.

The BCI system consisted of a headband with mounted dry EEG sensors (manufactured by Neurosky, Inc) that transmitted EEG readings to the computer through Bluetooth-enabled protocol. The headband was worn around the forehead, with a grounding reference electrode clipped to the earlobe (see Figure 2). Two dry EEG electrode sensors positioned to detect the EEG pattern from the frontal sites FP1 and FP2 were mounted on a headband. The advanced signal processing techniques in the brain-computer interface can pick up useful information about attentional activities from the frontal EEG recorded at sites Fp1 and Fp2 [12].

The possible effects of noise or artifacts such as extraocular activity on the EEG were considered and reduced in the BCI system. Since the noise and artifacts were generally uncorrelated with the attentiveness and in-attentiveness conditions, they were filtered out in our machine learning algorithm that extracted only discriminative features from EEG between the two conditions. To further reduce the electrooculography artifacts, we added a virtual EEG channel, which was the differential potential between Fp1 and Fp2. As a result, the system was not affected by normal eye movements.

#### Calibration

Prior to playing the video game (CogoLand), which was the main training activity, each participant underwent individual calibration using a colour Stroop task on the BCI-based attention training game system. During calibration, the participant performed the colour Stroop task to develop an individualized EEG profile of the optimal attentive state. The colour Stroop task required one to use the mouse to click on the name of the colour in which a word was spelt, and not the colour that the word spelt. The BCI-based attention training game system analyzed the critical EEG parameters during the correct attempts, compared to that when the participant was relaxing, to derive an individualized EEG pattern representing the participant’s most attentive state [12].

#### Playing the game

A computerized 3D graphic game, CogoLand was developed specifically as the training game. In CogoLand, the participant controlled an avatar via the signals detected by the EEG electrodes. This was computed into a BCI ADHD Severity Measure, or BASM (see next section for details of its computation). The BASM was then transformed to a score ranging from 0 (minimum attention) to 100 (maximum attention), which was reflected on the computer screen. The participant would hence need to ‘concentrate’ in order to move the avatar, which would move at a speed proportional to the participant’s attention level as measured by the BCI-based attention training game. The ‘higher’ the concentration level of the participant was, the higher would be the speed of the avatar’s movement. There were three difficulty levels in CogoLand. The main goal of the first level was to make the avatar run around an island in the shortest time possible. The next two levels had an additional component where the child needed to collect a series of fruits floating in the air as the avatar navigated through a pre-determined route in a colorful town. The child would use a specific key on the keyboard to make the avatar jump to collect the fruits which would appear along the journey. The child was asked to collect as many fruits as possible within a given timeframe, after which the number of fruits collected was entered into a personal logbook. At the third level the child had to collect the fruits in an order presented on the screen. A short break was allowed between attempts. For each training session, the individual would complete 30 minutes of training, including the breaks.

**Table 1:** ADHD rating scale IV (ARS-IV) inattentive (IA), hyperactive-impulsive (HI) and combined symptoms (COM) total raw scores as rated by parents*.

		Inattentive (IA)	Hyperactive-Impulsive (HI)	Combined (COM)
Week 0	Sample Size	19	19	19
	Mean (SD)	17.7 (5.0)	15.6 (3.9)	33.4 (7.8)
Week 8	Sample Size	19	19	19
	Mean (SD)	13.1 (5.0)	10.9 (4.4)	24.1 (8.5)
Week 20	Sample Size	17	17	17
	Mean (SD)	13.6 (4.5)	10.2 (5.1)	23.8 (8.9)
Week 24	Sample Size	17	17	17
	Mean (SD)	12.6 (3.4)	10.5 (4.3)	23.1 (6.9)

* The improvement compared to baseline scores in all domains was statistically significant.

#### BCI ADHD severity measure (BASM)

All raw EEG data obtained during calibration with the Stroop task was analyzed by the BCI system. It was screened to detect any abnormality in the EEG recordings, such as disconnected electrodes and saturated digital samples. Any abnormal EEG readings, including the readings within two seconds from the occurrence of the abnormality were excluded from analysis. The system then extracted discriminative rhythmic power features from the screened EEG using spatial-spectral filtering.

**Figure 3:**
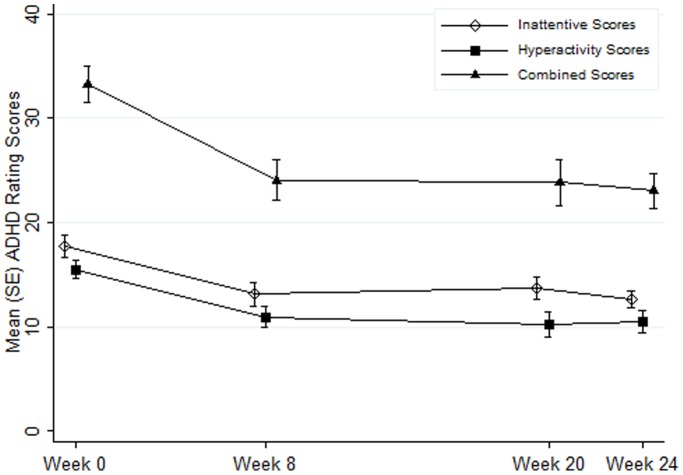
Mean ADHD Rating Scale IV (ARS-IV) Scores as rated by parents.

**Figure 4:**
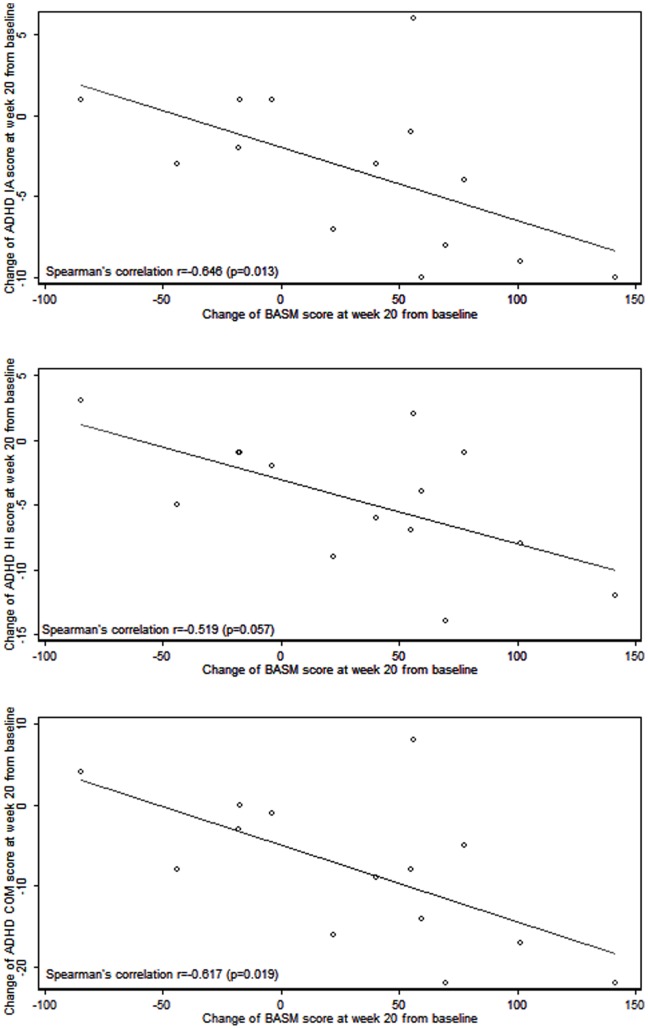
Correlations between change in BCI and BCI ADHD Severity Measure (BASM) scores from Week 0 to 20.

The system examined an array of 8 frequency bands, continuously covering from 4 Hz to 30 Hz. This arrangement not only covered traditional EEG bands from theta to beta, but also had a finer grid of frequency bands. Band powers were computed using the following procedure. First, the EEG data was segmented into a continuous sequence of 2-second long time blocks; in each block, power spectrum was computed using a 256-point Fast-Fourier-Transform technique; a specific band power was calculated as the sum of the spectrum powers at all the discrete frequencies in the band; and the specific band power was the average value of the band power over the time blocks. The band power was calculated for each EEG channel separately, in addition to the differential potential between the two channels. The BCI system then selected the band power features for maximizing the separation between attentive and inattentive states according to the information theory. A regression function would be applied by the BCI system to transform the selected features into a BASM score, which represented the severity of the inattentive symptoms of ADHD at the time of EEG recording. The BASM score was inversely proportional to the severity of the inattentive symptoms and the lower the BASM score, the more inattentive the individual was.

#### Treatment program

We used this BCI-based attention training game system for our intervention program (the BCI-based attention training program), which comprised of an intensive phase with 3 training sessions weekly for 8 weeks, followed by a maintenance phase with once-monthly booster training sessions for 3 consecutive months. At the end of every alternate training session starting from the second session, each participant would complete 2 short English and Mathematic worksheets consisting of multiple choice type questions on the computer. These worksheets were appropriate to their educational level and took approximately 10 minutes to complete. Each participant was instructed to concentrate like when they were playing the games, and their EEG was monitored during this period. Treatment was administered by 3 therapists trained to fit the headband and administer the BCI-based training program. All the therapists had obtained at least a graduate degree in psychology, and they administered treatment according to a standardized treatment protocol. Calibration was done at weeks 0, 4 and 8.

### Study Outcome Measures

At baseline, parents completed the 18-question ADHD Rating Scale, 4^th^ edition (ADHD-RS) [13]. The ADHD-RS was based on the DSM-IV criteria for ADHD and consisted of nine inattentive and nine hyperactive-impulsive symptoms, with a four-point scale (0 = never [less than once a week], 1 = sometimes [several times a week], 2 = often [once a day], and 3 = very often [several times a day]). Three measures were taken from the ADHD-RS: inattentive (IA) score (0–27), hyperactive-impulsive (HI) score (0–27) and combined (COM) score (0–54). The ADHD-RS was completed again at the end of weeks 4, 8, 20 (post-boosters), and 24. The primary outcome measures were the changes in ADHD-RS at weeks 8 and 20, compared to the baseline, to examine the efficacy of the intensive training and booster training sessions respectively. Additionally we also collected EEG information during each training session to examine for any significant EEG change.

## Results

### Study Completion and Dropout

There were 17 (85%) subjects who completed the entire study. One boy dropped out before 4 weeks, as the parent felt there was no improvement in the child’s behaviour. Two other boys dropped out between 4 and 8 weeks due to difficulty adhering to the treatment schedule.

### ADHD Rating Scale – IV Results

We conducted Intention-To-Treat (ITT) analyses on the results and excluded the subject who dropped out before week 4 as there was no follow up data at all. We carried forward the last available observation (behavioural rating) where appropriate. Multiple imputations using Markov chain Monte Carlo and a per protocol analysis including only subjects who completed the study were also conducted and the results did not show much difference from that based on the last observation carried forward method.

Table 1 summarizes parent-rated ADHD-RS scores at various study visits for the participants included in the analysis. No deviation from normal distribution was found for the ADHD-RS scores using both normality tests and graphic methods. Changes of these scores at week 8 and 20 from baseline were assessed by the paired t-test. Similarly, mean changes in these scores at week 20 from week 8 and changes at week 24 from baseline were analyzed to determine any booster effect and long-term effect, respectively. At week 8, the mean (SD) change compared to week 0 for inattentive (IA) symptoms was −4.6 (5.9) and the median (range) change was −3.0 (−17.0, 4.0). It was shown that this median change was statistically significant (p = 0.003). Similarly, the mean changes in parent-rated hyperactive impulsive (HI: mean change = −4.7 (5.6)) and combined (COM: mean change = −9.3 (11.0)) symptoms were statistically significant (p = 0.002for both HI and COM).

There was no statistically significant change in parental observation of inattentive and hyperactive-impulsive symptoms on the ADHD-RS at 20 weeks compared to 8 weeks, or at 24 weeks compared to 20 weeks. When examining the ratings at 24 weeks, compared to the baseline score, there was significant improvement in parent-rated inattentive and hyperactive-impulsive symptoms (mean changes = −5.0 (5.8) and −5.7 (5.1) respectively and p≤0.01 for both IA and HI). These results appear to suggest that monthly booster training for 3 consecutive months after an intensive 8-week training did not significantly improve inattentive or hyperactive-impulsive symptoms further. The behavioural benefits from the intensive training at 8 weeks were sustained at 24 weeks. The child’s age and gender did not have any statistically significant effect on ADHD-RS scores in this study.

Figure 3 is a graphical representation of the change in mean and standard error of the ADHD-RS scores as rated by parents over the 24 weeks duration of the study.

### EEG Results

We used EEG data from the calibration/re-calibration sessions (at week 0 and 20) and examined the BASM scores. We had to exclude the 3 participants who dropped out before week 8 and 3 other patients who had missing EEG data at Week 20. Thus, we analyzed a total of 14 participants from the original 20 recruited. When comparing the BASM scores at Week 0 and at Week 20, there was an increase in the mean score (standard deviation) from 60.9 (81.0) to 96.9 (64.7), although paired t-test showed that the change was not statistically significant (mean change = 32.5 (60.8), p = 0.067).

### Predictors of Clinical Outcome

Linear regressions showed that baseline IA, HI and COM scores statistically significantly predicted their respective changes of ADHD rating scale scores from week 0 to 8 (β (SE) = −0.7 (0.2), −0.9 (0.3) and −0.9 (0.3) respectively and p = 0.013, 0.008 and 0.007 respectively). Thus, a higher score on the ADHD-RS at baseline predicts greater improvement at week 8. We investigated the possible correlations between the change in BASM scores and the changes in scores on ADHD rating scale from Week 0 to Week 20. Correlation analyses performed in Figure 4 showed that there were strong negative correlations between changes in these two scores (Spearman’s correlation coefficients were −0.646, −0.519, and −0.617 for IA, HI and COM respectively). This was statistically significant for both IA (p = 0.013) and COM (p = 0.019), but not for HI (p = 0.057). In other words, increasing BASM scores was associated generally with decreasing ADHD scores. We did not find age, gender or ADHD subtype to predict the ADHD-RS changes at weeks 8 or 20.

### Adverse Events

The intervention was also well tolerated. In this trial, the main side effect reported was headache, which affected two of the participants. The severity of the headache was reported to be mild and did not stop them from continuing with treatment.

## Discussion

The present study evaluated the new version of the BCI-based attention training program, which included dry sensors and blue tooth technology in place of EEG leads with a game CogoLand, in the treatment of combined and inattentive subtypes of ADHD. Our results show that an 8-week intervention significantly improved inattentive symptoms of ADHD, based on a behavioural rating scale by parents. Among children with the combined subtype of ADHD, parents also reported a significant improvement in their hyperactive-impulsive symptoms on the ADHD Rating Scale. When these children received monthly training sessions subsequently, the behavioural improvements were sustained but did not further improve. Those with more severe symptoms were also the ones who showed greater improvement.

This built upon our initial report on the training program’s positive effects on treating ADHD symptoms. Although shown to improve inattentive symptoms more than hyperactive-impulsive symptoms then, we now found significant treatment effects for both inattentive and hyperactive-impulsive symptoms of ADHD. It is possible that non-specific factors like behavioural contingencies, self efficacy and a structured training environment could have contributed to improvement in the hyperactive-impulsive symptoms. Children with both inattentive and combined subtypes, which are far commoner than the hyperactive-impulsive subtype, appear to improve with treatment.

Additionally we also found that the change in BASM score correlated with the change in behavioural rating score by parents. This provided some evidence that the improvement with training, as reflected by the BASM score, might explain the improved ADHD symptoms reported by parents. The BASM score also appeared to be a good surrogate marker for observed inattentive behaviour. In many ways this is a refinement from previous work that looked at specific EEG bands. Neurophysiological studies have previously shown that children with ADHD exhibit specific patterns on the electroencephalogram (EEG) and this has been utilized clinically to diagnose, treat and even predict response to treatment with medication [14], [15], [16], [17], [18]. EEG studies of children with ADHD showed the majority to exhibit abnormal patterns of resting cortical activity including increased slow-wave activity (primarily theta waves), decreased fast-wave activity (primarily beta waves) and increased beta-theta ratio [19], [20], [21]. These findings are consistent with the inattentive symptoms exhibited in ADHD, as beta activity is associated with concentration or mental activity whereas theta activity is associated with drowsiness [22]. During the performance of cognitive tasks, children with ADHD exhibit EEG changes similar differences compared to normal matched controls [23], [24], [25]. Through childhood EEG, it was also possible to predict those at risk of having ADHD symptoms which persisted into adulthood [26]. Further research is therefore warranted to elucidate the neural mechanisms explaining the observed behavioural improvement.

BCI-based attention training game system can offer several advantages over current evidence-based treatment options offered in most clinical practices. It has less adverse events compared to medication. Unlike behavioural management or parent training, there is no need for regular clinic visits which can be inconvenient. The system potential utility adds to the growing research in an area that was a precursor to this approach namely neurofeedback. Computer-based neurofeedback attention training programmes have been shown to improve inattentive symptoms [27], [28]. Neurofeedback therapy was developed based on the knowledge that children with ADHD exhibited specific EEG patterns, and EEG feedback training directed at normalizing these rhythms might yield sustaining clinical benefits [4]. This approach was shown to be efficacious in treating ADHD in several trials [15], [29], [30]. When used in combination with other standard modalities of treatment, additional behavioural improvement can also be observed [31]. Neuro-imaging studies show that neurofeedback therapy results in functional normalization of the brain systems mediating selective attention and response inhibition in children with ADHD [32].

There are some important limitations to our present study. This is an uncontrolled open-label trial and thus the parents who completed the behavioural rating scale were not blinded. This could have resulted in an exaggerated treatment effect. Unfortunately the non-response rate from the children’s schoolteachers was too high for the results to be interpreted meaningfully. A well-designed randomized controlled trial is needed to evaluate the efficacy of BCI-based attention training program in treating ADHD.

### Conclusion

Brain computer interface based attention training game can be a potential new treatment for ADHD. A randomized controlled trial to study the efficacy of this intervention and the neural mechanisms underlying the behavioral improvements is currently underway. It represents a novel treatment modality for ADHD, which not only has the potential for being used in combination with present evidence-based treatment, but also uniquely in a non-clinical setting.

## Supporting Information

Protocol S1 **Trial protocol.**
(PDF)

Checklist S1 **CONSORT checklist.**
(DOC)
