# Supplementary material for: A Brain-Computer Interface Based Attention Training Program for Treating Attention Deficit Hyperactivity Disorder
Source: PLoS One. 2012 Oct 24;7(10):e46692. doi: 10.1371/journal.pone.0046692 (PMC3480363; doi:10.1371/journal.pone.0046692)
Supplement: Protocol S1 — Trial protocol. (PDF) [file pone.0046692.s001.pdf]

**Study Reference Number: 2009/00395**

**Version Number: 1**

**Please select the appropriate form for submission to the DSRB. Please refer to the explanatory notes below if you need more information.**

- ◉ DSRB Application Form 1 - Non Exempt Category
- ○ DSRB Application Form 2 - Exempt Category

**Research activities in which the only involvement of human subjects will be in one or more of the following categories may be able to qualify for the Exempt category.**

**Please click on the DSRB Application Form 2 - Exempt Category option above to view the categories.**

**DSRB Application Form 1 - Non Exempt Category**

**Principal Investigators should use Application Form 1 if their research activity does not qualify under the Exempt Category. Application Form 1 should be used for submissions for the Full Board Review and Expedited Review.**

**DSRB Application Form 2 - Exempt Category**

**Research activities in which the only involvement of human subjects will be in one or more of the following categories may be able to qualify for the Exempt category.**

**IMPORTANT: The criteria for the Exempt category do not apply when the research activity:**

**(i) involves prisoners**

**(ii) involves children, when the research involves survey or interview procedures or observations of public behavior, except when the investigator(s) do not participate in the activities being observed**

**(iii) is a US FDA-regulated research activity.**

**A1 Please enter the full title for this study.**

A Randomised Controlled Trial of a Brain-Computer Interface Based Intervention for the Treatment of ADHD

**A2 Study Administrators are persons who are responsible for administrative matters related to the Study. They can be the Study Coordinators, Research Nurses or Clinical Research Associates, and need not be part of the Study Team.**

**While the Principal Investigator remains the primary contact person, the DSRB may contact the Study Administrators for clarification of administrative matters related to the Study.**

**Study Administrators may also assist the PI in completing the various online forms and reports, however, only the PI may 'submit' these online forms and reports to the DSRB.**

**This section is optional but PI's are encouraged to nominate at least one Study Administrator. You may assign Study Administrators for this study below.**

| No. | Name | Institution | Department | Role | Email |
|---|---|---|---|---|---|
| 1 | Shi Jie, Nikki Lim | Institute of Mental Health | Child & Adolescent Psychiatry | Study Administrator | Nikki_SJ_LIM@ imh.com.sg |
| 2 | Anushia Panchalingham | Not Applicable (Non-NHG) | Not Applicable (Non-NHG) | Study Administrator | anushia.lingham@ scri.edu.sg |
| 3 | Stephanie Sze Wei Teng | Institute of Mental Health | Child & Adolescent Psychiatry | Study Administrator | Stephanie_SW_TENG@ imh.com.sg |

## B1 Study Sites & Study Team Members

**All investigators who have a responsibility for the consent process and/or direct data collection for this study should be listed below.**

**Study Team Members with registered user account with us will be notified of their participation in this study when the Application is submitted.**

**For a Multi-centre study, please appoint a Site PI for each site (Mandatory).**

**The Principal Investigator will be the Site PI for their own Institution, and will also be the primary contact person for the DSRB.**

**(i) 'Overall Principal Investigator': Lim Choon Guan**

**(ii) Study Sites under the oversight of NHG DSRB (eg: NHG's Institutions, St Luke's Hospital, HSA, Dover Park Hospice, etc)**

| No. | Study Site | Name | Study Role | Institution | Department | Min Training |
|---|---|---|---|---|---|---|
| 1 | Institute of Mental Health | Guan Cuntai | Co-Investigator | A*STAR | Institute for Infocomm Research | Not completed |
| 2 | Institute of Mental Health | Lim Shih Hui | Co-Investigator | National Neuroscience Institute | Neurology | Not completed |
| 3 | Institute of Mental Health | Krishnan, Ranga R. | Co-Investigator | Duke-NUS Graduate Medical School | | Not completed |
| 4 | Institute of Mental Health | Choon Guan Lim | PI | Institute of Mental Health | Child & Adolescent Psychiatry | Completed |
| 5 | Institute of Mental Health | Zhao Yudong | Co-Investigator | Singapore Clinical Research Institute | Biostatistic | Not completed |
| 6 | Institute of Mental Health | Cheung Yin Bun | Co-Investigator | Singapore Clinical Research Institute | Biostatistic | Not completed |
| 7 | Institute of Mental Health | Daniel Fung | Co-Investigator | Institute of Mental Health | Child & Adolescent Psychiatry | Completed |
| 8 | Institute of Mental Health | Lee Tih-Shih | Co-Investigator | Duke-NUS Graduate Medical School | | Not completed |

**(iii) Other external Study Sites under the supervision of the 'Overall Principal Investigator' (eg: Nursing Home, Community Hospitals, Public Community etc)**

**B2 External Study Site (for Institutions NOT under the oversight of NHG DSRB)**

**(i) Are there any other independent study sites by another PI which are conducting the same study?**

○ Yes

◉ No

**B3 Research Specialty**

**Please indicate the Primary Specialty.**

| No. | Primary Specialty | Primary Sub Specialty |
|---|---|---|
| 1 | Psychiatry | Addiction Medicine |

**Please indicate/add Secondary Specialties.**

| No. | Primary Specialty |
|---|---|
| There is nothing. | |

B4

**i. Which Domain Specific Review Board (DSRB) is this application being submitted to? DSRB Domain A**

**ii. Has the study been submitted to another IRB?**

◉ No
○ Yes

**iii. Has the application been previously rejected by any IRB? (Including NHG-DSRB)**

◉ No
○ Yes

**The Conflict of Interest Declaration section must be completed by the PI on behalf of the Study Team if any member of the Study Team has any potential conflicting interest while conducting the research. Any such member(s) must complete and submit their Declarations when this application is submitted.**

**Conflicting Interest - A conflicting interest can be broadly defined to refer to any interest of the investigator that competes with the investigator's obligation to protect the rights and welfare of research subjects.**

**Financial Interest - Significant Financial Interest means anything of monetary value, including but not limited to, salary or payments for services (e.g. consulting fees or honoraria); equity interests (e.g. stocks, stock options or other ownership interests); intellectual property rights (e.g. patents, copyrights and royalties from such rights), and board or executive relationships.**

**The Conflict of Interest Declaration Section must be submitted to the DSRB via protocol amendments if any of the circumstances relevant described herein change during the conduct of the research.**

**Choon Guan Lim**

○ Yes
◉ No

**D Please select a category that best describes your application. Please note that for clinical trials, additional questions are applicable. Please take some time to choose carefully.**

Clinical Trials

**Choose this if your research involves:**

**(1) Administering a drug, device, or biologic as part of the research intervention, or**

**(2) Performing surgical procedures as part of research intervention**

Questionnaire/ Survey/ Interviews

**Choose this if your research involves:**

**(1) Administering questionnaires/surveys/interviews. This type of research may also include a medical records review component.**

Medical Records Review

**Choose this if your research involves:**

**(1) Collection of data for a specific research project by review of medical records including results of routine diagnostic tests performed for standard clinical purposes**

**(2) Prospective and/or retrospective data collection**

Clinical Research

**Choose this if your research involves:**

**(1) Collection of blood by venepuncture, finger stick, etc.**

**(2) Prospective collection of biological specimen by invasive or non invasive means including biopsies, FNAC's, fundoscopy etc.**

**(3) Collection of data through research procedures such as X rays, MRI, ultrasound, ECG, EEG, etc.**

**(4) Any other research categories.**

**D1 Please select one category that best describes your research activities.**

- ◉ Clinical Trials (which includes Drug, Device and Surgical-Procedure Trials)
- ○ Questionnaire/ Survey/ Interviews
- ○ Medical Records Review
- ○ Clinical Research

What does the study involve?

- ○ Drug/Biologic
- ◉ Device
- ○ Surgical Procedure

Note: Clinical Trial Certificate from Health Sciences Authority might be required if you are testing the safety and efficacy of the medicinal product. You should check with HSA if you are unsure.

**D2 Is this a US FDA IND/IDE study or data is intended to be reported to FDA in support of a IND/ IDE application?**

- ○ Yes
- ◉ No

**Note: US FDA-regulated (IND) research activities cannot qualify for Exemption from DSRB Review and Waiver of Informed Consent. The application must be submitted using the Non-Exempt Application Form.**

**D3 Is this study subjected to any of the following regulations:**

- ○ No

○ Yes

☐ US Code of Federal Regulations 45 CFR 46
☐ US Code of Federal Regulations 21 CFR 50
☐ US Code of Federal Regulations 21 CFR 56
☐ US Code of Federal Regulations 21 CFR 312
☐ US Code of Federal Regulations 21 CFR 812
☐ Others

**E1 Who will be responsible for the payment and compensation of injury or illness arising from participation of subjects in the study?**

**(Note: For investigator - For investigator-initiated studies - Contact your OBR/CRU for more information on available NHG Clinical Trial Compensation Insurance Scheme.)**

The hospital/National Healthcare Group. The intervention involved in this is non-invasive and it is not expected to result in any form of injury.

**E2 Please give information regarding the study's Funding source or Sponsor information.**

- ○ No funding is required for this study to be carried out
- ○ Pharmaceutical / Industry Sponsored
- ◉ Grant

**i. Name of Grant Agency and Grant Name Others**

**Please specify: A*STAR**

**ii. Grant amount applied for 1400000.0**

**iii. Date of Grant application deadline 04-Jun-2009**

**iv. Has the Grant application been approved?**

- ○ Yes. Grant application successful.
- ◉ No. Grant application is pending approval.

**Is the study's initiation dependent on Grant approval?**

- ◉ No. The study can be started without the Grant.
- ○ Yes. The study is dependent on the Grant to start.

**E3 Who will be responsible for research-related costs? For sponsored studies, please list the costs that will be borne by the sponsor. You may wish to attach the Financial Agreement / Clinical Trial Assurance if it is available.**

We have obtained In-principle approval for Flagship funding from Exploit Technologies A*Star to fund this study.

**F1 Please provide an abstract of your proposed research (Up to 300 words).**

**Your abstract must contain:**

**Aims**

**Methodology**

**Importance of proposed research to science or medicine**

**Potential benefits & risks**

AIMS:This study aims to examine the efficacy of a brain-computer interface system for the treatment of inattentive symptoms of Attention Deficit Hyperactivity Disorder (ADHD).METHODOLOGY:SELECTION OF SUBJECTS This study will enroll 270 children aged 6 to 12 newly referred to the Child Guidance Clinic for ADHD who have never received treatment with medication. An intake (baseline) assessment will then be done. Either of the child's parents or legal guardian will be interviewed. The child should satisfy the following criteria for the diagnosis of ADHD:1. DSM-IV-TR criteria for ADHD, either the combined or inattentive subtype, based on clinical interview2. Diagnostic Interview Schedule for Children (DISC) They should not have any of the exclusion criteria. The clinic's psychiatrists will refer interested patients, whose parents have opted for their children not to take medication for ADHD. Exclusion criteria:1. Present or history of medical treatment with stimulant medication and Atomoxetine2. Co-morbid severe psychiatric condition or known sensori-neural deficit e.g. complete blindness or deafness (such that they cannot play computer games)3. History of epileptic seizures4. Known mental retardation (i.e. IQ 70 and below)5. Predominantly Hyperactive/impulsive subtype of ADHD (i.e. no predominant inattentive symptoms)Informed consent will be obtained from the parents, and assent from the subjects, prior to any form of assessment or intervention as part of the study. The details of the study will be explained to both the child and the parents, and they will be invited to participate. Those who are interested will be scheduled for a baseline assessment visit at the clinic. The investigator will allow all subjects to have sufficient time to contemplate and ask any queries pertaining to the informed consent.Subjects in both intervention and control groups will continue to receive care from their treating psychiatrists. Given that the parents would already have opted not to have their child receive medication, the main modality of treatment available will be psychosocial intervention.EXPERIMENTAL DESIGNThis project will be carried out in 2 phases:Phase 1: Pilot StudyA small group of 20 children will be recruited for the pilot study. The aim of the pilot study is to test out the new protocol and to ensure that it is not mentally traumatic to the children. All subjects will undergo the same intervention and assessments as the children in the first 8 weeks of the phase 2 efficacy study. There will be no control group and no booster sessions in the pilot study.Phase 2: Efficacy Study250 children with ADHD who have not received and have declined treatment with medication will be randomised into either the intervention or the control group. Randomisation will be stratified for gender and will be balanced within blocks. Both groups will receive concurrent behavioural management for ADHD. It is expected that the male:female ratio will be approximately 9:1. They will be randomised into either the intervention or control group, giving 125 subjects in each group, with stratification by gender.INTERVENTION GROUPThe 125 subjects in the intervention group will take part in a total of 24 sessions spread over a 8-week period, followed by one-monthly booster sessions for another 3 months. Each subject will first need to master a simple concentration task. After this, the child will go on to play a series of training tasks. Each training activity employs the BCI system, and is controlled by the child's concentration, which is fed to the computer via the EEG leads. The child watches the activities on a computer screen. Each session takes approximately thirty minutes. In addition, as part of the treatment protocol, they will be given worksheets appropriate for their educational level (1 Maths and 1 short comprehension exercise) at the end of every alternate intervention over the 8-week period, and during each of the three monthly 'booster' sessions. Their EEG activity will be recorded during these tasks. Subjects will be advised to concentrate during the tasks just as they did during the BCI intervention. This will train the child to transfer their 'concentration skill' learnt during the BCI training to their everyday task (homework forms a major component). CONTROL GROUPThe 125 subjects in the control group will not receive BCI training during the first 8 weeks of their study participation; they will act as wait-list controls. At week 9, subjects in the control group will go through the BCI training similar to the intervention group. They will take part in a total of 24 sessions spread over an 8-week period, followed by one-monthly booster sessions for another 3 months. They will also be given worksheets appropriate for their educational level (1 Maths and 1 short comprehension exercise) at the

end of every alternate intervention over the 8-week period, and during each of the three monthly ‘booster’ sessions. Their EEG activity will be recorded during these tasks. They will be followed-up for one month. Their participation in the research study will last 8 months. The subjects in the control group will complete the exact same questionnaires at the same time-points as the intervention group. ASSESSMENTAt baseline, the parent/ legal guardian will complete the following questionnaires:1. ADHD Rating Scale2. Child Behaviour Checklist (CBCL)The child will undergo the following assessments:1. Wechsler Individual Achievement Test, 3rd edition or WIAT-III, to assess academic achievement The following questionnaires will be mailed to the child’s form teacher in school. They will then be returned to the Principal Investigator in a self-addressed envelop.1. ADHD Rating Scale2. Teacher’s Report Form (TRF)At baseline assessment, children in both intervention and control groups will play a concentration game and be measured for EEG activity.The ADHD Rating Scale will be administered to the parents and teachers of all subjects in both study groups at weeks 4, 8, 12, 16, 20, 24, 28, and at 3 months post-intervention (week 32):The following will be completed at weeks 8, 20 and 32:1. Child Behaviour Checklist (CBCL): parent2. Teacher’s Report Form (TRF): teacher3. DISC-IV (week 8 and 20 only)4. WIAT-III (at week 20 only)The subjects and their parents will make clinic visits for these scheduled assessments. All questionnaires to be completed by the teachers will be mailed to the child’s school. They will then be returned to the Principal Investigator in a self-addressed envelop. Reminders will be sent at 14-day intervals if the teachers did not return the questionnaire in time. At weeks 0, 4, 8, 12, 16, 20, 24, 28 and 32, a clinician (who is blinded to the group assignment) will also complete the following: 1. Clinical Global Assessment Scale2. Clinical Global Impression - Severity Scale3. Clinical Global Impression – Improvement ScaleThe primary outcome will be change in the inattentive score on the ADHD Rating Scale as rated by teachers at week 8. Teachers’ ratings are preferred as they are blind to the intervention received by the child. Secondary outcomes include inattentive score rated by parents on the ADHD Rating Scale at weeks 8 and 20, ADHD diagnosis status based on DISC, CBCL/TRF attention problem scores, clinician rated C-GAS and CGI scores and academic assessment.STATISTICAL CONSIDERATIONSBased on a moderate effect size of 0.4 on ADHD Rating Scale inattentive score improvement rated by teachers, power of 80% and type I error of 5%, a sample size of approximately 115 subjects per group is necessary. This includes an allowance for up to 25% losses to follow-up. Intervention groups will be compared for balance in baseline characteristics and in CONSORT flow chart. The primary outcome will be improvement on the ADHD Rating Scale scores at completion of the 20-session intervention. The main analysis will be based on two-sample t-test. Continous Secondary outcomes will be analyzed by t-test or Mann-Whitney U test where approprieate. Chi-square test or Fisher’s exact test will be applied for categorical secondary endpoints. Subjects will be considered as ‘drop-outs’ if they:1. Become lost to follow-up or discontinues intervention at any point.2. Receive any medication (e.g. methylphenidate, atomoxetine, tricyclic anti-depressant) or supplement (e.g. omega-3 fatty acid) which can improve ADHD symptoms during the trial period. The parent/child will be asked about this each time they turn up at the clinic.POTENTIAL DIFFICULTIESSubjects may have difficulties making their way down to the clinic for the frequent interventions. As far as possible, we will attempt to avoid involving examination periods (May and October) during the 8-week intervention.INTROIMPORTANCE:Therapy using non-invasive BCI system-based games may represent one alternative means to treat children with ADHD symptoms.
If demonstrated to be efficacious, parents are likely to prefer this as an alternative to current treatment modalities of medication (with potentially serious side effects) and behaviour management (parents generally find this difficult and challenging).POTENTIALBENEFITS:All subjects will eventually receive Brain Computer Interface (BCI) intervention. These subjects may see improvement in their attention.POTENTIALRISKS:From literature, the potential risks may include seizure or destabilization of the individual's mood state. To minimise such risk, individuals with seizure or severe psychiatric disorder will be excluded from participation.

**F2 What are the Specific Aims of this study?**

This study aims to examine the efficacy of a brain-computer interface system for the treatment of inattentive symptoms of Attention Deficit Hyperactivity Disorder (ADHD).This study aims to examine the efficacy of a brain-computer interface system for the treatment of Attention Deficit Hyperactivity Disorder (ADHD). Our hypothesis is that children with ADHD receiving treatment with the BCI-based training will improve to a greater extent than the control group.

**F3 What is the Hypothesis of this study?**

Our hypothesis is that children with ADHD receiving treatment with the BCI-based training will improve to a greater extent than the control group.

**F4 Please briefly describe the background to the current study proposal. Critically evaluate the existing knowledge and specifically identify the gaps that the proposed study is intended to fill.**

Attention-deficit/hyperactivity disorder (ADHD) is the commonest psychiatric condition among children and adolescents seen at the Child Guidance Clinic (CGC) in Singapore, much like the rest of the world. ADHD is diagnosed clinically and is characterised by symptoms of inattention and/or hyperactivity/ impulsivity, with onset in childhood. (1) Community studies have found the prevalence of ADHD to be between 1.7% and 16% (2). Recent studies demonstrated that up to half of the children with ADHD continue to have symptoms in adulthood (1,3). Male-female ratios range from 9:1 to 6:1 in clinical samples but are about 3:1 in community-based population studies (4). ADHD is associated with academic underachievement, work difficulty, social rejection, driving accidents, smoking, alcohol and drug abuse, and poor self esteem. In view of the prevalence, persistence and long term impairment, ADHD is an important public health problem. About 500 new cases of ADHD are referred to CGC annually for treatment, and the number is still increasing.Management of ADHD includes pharmacological and behavioural management. Pharmacological treatment includes stimulant and non-stimulant medications (1,5). Methylphenidate is the only stimulant medication available in Singapore. Parents are often concerned about its side effects including poor appetite, physical growth retardation and cardiovascular effects. Being a potential drug of abuse, methylphenidate is also avoided in patients who may be likely to abuse the medication. On the other hand, atomoxetine, a relatively new non-stimulant medication, is expensive with significant side effects. Atomoxetine carries a 'black box label' warning of potential increased suicidal thinking associated with its use. Behavioural management, though important, has been shown to be less superior than pharmacological treatment (6). Follow-up results of the NIMH Collaborative Multisite Multimodal Treatment Study of Children With Attention-Deficit/Hyperactivity Disorder (MTA) showed that despite initial symptom improvement during treatment, the superiority of medication was lost by 3 years, and the 8-year follow up showed that children with combined-type ADHD exhibit significant impairment in adolescence (7). Brain-computer interface (BCI) is a direct communication pathway between a human brain and an external device. It is a technology that enables people to interact with computers through their thoughts. Electroencephalogram (EEG) is the best studied non-invasive interface facilitatating such communication, mainly due to its fine temporal resolution, ease of use, portability and low set-up cost. It presents the user with realtime feedback on brainwave activity, as measured by electrodes on the scalp, typically in the form of a video display, sound or vibration (8). In children with ADHD, reduced levels of the higher-frequency brain waves are especially noticeable in the prefrontal cortex, an area involved in attention control. They also have an increase in lower-frequency waves, especially theta waves from 4 to 7.5 Hz. Neurofeedback, a form of biofeedback programme (9,10,11), has been used in a limited fashion but is not well studied.We have developed a series of BCI-based training activities incorporating our patented attention detection technology into interactive games for the treatment of ADHD. As the BCI extracts a quantifiable attention level from EEG, users can employ their attention to play these games directly. Therefore, there is no need for the users to follow any prompt or output on the screen; in other words, this works in an 'asynchronous' mode. With such a mode, user can start to play the game straightaway, without any learning. At the same time, since the user can control a game without any feedback (auditory or visual), there is a unique feed-forward mechanism as well. To help the user progress through the series of activities of increasing difficulty, players will first need to master a simple concentration task before moving on to the next task according to the treatment protocol. Most therapy involves at least a total of 20 sessions (11); as such, our intervention protocol includes 20 sessions followed by 3 one-monthly 'booster training'. The treatment protocol also involves a homework task for every alternate session, during which the child will be 'trained' to use their newly-acquired concentration skill during everyday tasks. Our intervention and treatment protocol requires little training for the therapist, and the equipment required is simple and portable.This treatment modality, if efficacious for treating ADHD, is likely to be more acceptable than medication or behavioural treatment to parents. Children themselves are also more likely to enjoy the treatment and hence, comply better. In the longer term, this approach and system may have potential for attentional and cognitive enhancement for patients with cognitive impairment or early dementia.

**F5 Please provide a list of relevant references.**

1. American Academy of Child and Adolescent Psychiatry. Practice Parameters for the Assessment and Treatment of Children, Adolescents, and Adults with ADHD 1997. Updated 2007.2. Barkley RA, Fischer M, Edelbrock CS, Smallish L. The adolescent outcome of hyperactive children diagnosed by research criteria, I: an 8-year prospective follow-up study. J Am Acad Child Adolesc Psychiatry. 1990;29:546-557.3. Barkley RA, Fischer M et al. The persistence of attention deficit/hyperactivity disorder into young adulthood as a function

of reporting source and definition of disorder. J Abnorm Psychol 2002; 111(2):279-89.4. Biederman J, Mick E, Faraone SV, et al. Influence of gender on attention deficit hyperactivity disorder in children referred to a psychiatric clinic. Am J Psychiatry. 2002;159:36-42. 5. Eric Taylor et al: European clinical guidelines for hyperkinetic disorder – first upgrade. European Child amp; Adolescent Psychiatry, Vol. 13, Supplement 1 (2004)6. MTA Cooperative Group. National Institute of Mental Health Multimodal Treatment Study of ADHD follow-up: changes in effectiveness and growth after the end of treatment. Pediatrics 2004;113;762-769.7. Molina BS et al. The MTA at 8 years: prospective follow-up of children treated for combined-type ADHD in a multisite study. J Am Acad Child Adolesc Psychiatry. 2009 May;48(5):461-2.8. Hirshberg LM, Chiu S, Frazier JA. Emerging brain-based interventions for children and adolescents: overview and clinical perspective. Child Adolesc Psychiatric Clin N Am 2005;14;1–19.9. Friel, Patrick N. EEG Biofeedback in the Treatment of Attention Deficit/Hyperactivity Disorder. Alternative Medicine Review 2007;12;2;146-51.10. Monastra VJ. Electroencephalographic biofeedback (neurotherapy) as a treatment for attention deficit hyperactivity disorder: rationale and empirical foundation. Child Adolesc Psychiatr Clin N Am. 2005 Jan;14(1):55-82.11. Strehl U, Leins U, Goth G, Klinger C, Hinterberger T, Birbaumer N. Self-regulation of slow cortical potentials: a new treatment for children with attention-deficit/hyperactivity disorder. Pediatrics. 2006 Nov;118(5):e1530-40.

**F6 Please submit a copy of at least two relevant papers.**

**Monastra_White_Paper.pdf**

**EEG paper 2007.pdf**

**F7 Please state concisely the importance of the research described in this application by relating the specific aims to the long term objectives.**

Therapy using non-invasive BCI system-based games may represent one alternative means to treat children with ADHD symptoms. If demonstrated to be efficacious, parents are likely to prefer this as an alternative to current treatment modalities of medication (with potentially serious side effects) and behaviour management (parents generally find this difficult and challenging).

**F8 Discuss in detail the experimental design and procedures to be used to accomplish the specific aims of the study.**

SELECTION OF SUBJECTS This study will enroll 270 children aged 6 to 12 newly referred to the Child Guidance Clinic for ADHD who have never received treatment with medication. An intake (baseline) assessment will then be done. Either of the child’s parents or legal guardian will be interviewed. The child should satisfy the following criteria for the diagnosis of ADHD: 1. DSM-IV-TR criteria for ADHD, either the combined or inattentive subtype, based on clinical interview 2. Diagnostic Interview Schedule for Children (DISC) They should not have any of the exclusion criteria. The clinic's psychiatrists will refer interested patients, whose parents have opted for their children not to take medication for ADHD. Exclusion criteria: 1. Present or history of medical treatment with stimulant medication and Atomoxetine 2. Co-morbid severe psychiatric condition or known sensori-neural deficit e.g. complete blindness or deafness (such that they cannot play computer games) 3. History of epileptic seizures 4. Known mental retardation (i.e. IQ 70 and below) 5. Predominantly Hyperactive/impulsive subtype of ADHD (i.e. no predominant inattentive symptoms) Informed consent will be obtained from the parents, and assent from the subjects, prior to any form of assessment or intervention as part of the study. The details of the study will be explained to both the child and the parents, and they will be invited to participate. Those who are interested will be scheduled for a baseline assessment visit at the clinic. The investigator will allow all subjects to have sufficient time to contemplate and ask any queries pertaining to the informed consent. Subjects in both intervention and control groups will continue to receive care from their treating psychiatrists. Given that the parents would already have opted not to have their child receive medication, the main modality of treatment available will be psychosocial intervention. EXPERIMENTAL DESIGN This project will be carried out in 2 phases: Phase 1: Pilot Study A small group of 20 children will be recruited for the pilot study. The aim of the pilot study is to test out the new protocol and to ensure that it is not mentally traumatic to the children. All subjects will undergo the 8-week intervention programme with no boosters. They will receive the sameundergo the same intervention and assessments as the children in the first 8 weeks of the phase 2 efficacy study. There will be no control group and no control group and no booster sessions in the pilot study. Phase 2: Efficacy Study 250 children with ADHD who have not received and have declined treatment with medication will be randomised into either the intervention or the control group. Randomisation will be stratified for gender and

will be balanced within blocks. It is expected that the male:female ratio will be approximately 9:1. They will be randomised into either the intervention or control group, giving 125 subjects in each group, with stratification by gender. INTERVENTION GROUP The 125 subjects in the intervention group will take part in a total of 24 sessions spread over a 8-week period, followed by one-monthly booster sessions for another 3 months. Each subject will first need to master a simple concentration task. After this, the child will go on to play a series of training tasks. Each training activity employs the BCI system, and is controlled by the child's concentration, which is fed to the computer via the EEG leads. The child watches the activities on a computer screen. Each session takes approximately thirty minutes. In addition, as part of the treatment protocol, they will be given worksheets appropriate for their educational level (1 Maths and 1 short comprehension exercise) at the end of every alternate intervention over the 8-week period, and during each of the three monthly 'booster' sessions. Their EEG activity will be recorded during these tasks. Subjects will be advised to concentrate during the tasks just as they did during the BCI intervention. This will train the child to transfer their 'concentration skill' learnt during the BCI training to their everyday task (homework forms a major component). CONTROL GROUP The 125 subjects in the control group will not receive BCI training during the first 8 weeks of their study participation; they will act as wait-list controls. At week 9, subjects in the control group will go through the BCI training similar to the intervention group. They will take part in a total of 24 sessions spread over an 8-week period, followed by one-monthly booster sessions for another 3 months. They will also be given worksheets appropriate for their educational level (1 Maths and 1 short comprehension exercise) at the end of every alternate intervention over the 8-week period, and during each of the three monthly 'booster' sessions. Their EEG activity will be recorded during these tasks. They will be followed-up for one month. Their participation in the research study will last 8 months. The subjects in the control group will complete the exact same questionnaires at the same time-points as the intervention group. ASSESSMENT At baseline, the parent/legal guardian will complete the following questionnaires: 1. ADHD Rating Scale 2. Child Behaviour Checklist (CBCL) The child will undergo the following assessments: 1. Wechsler Individual Achievement Test, 2nd edition or WIAT-II, to assess academic achievement The following questionnaires will be mailed to the child's form teacher in school. They will then be returned to the Principal Investigator in a self-addressed envelop. 1. ADHD Rating Scale 2. Teacher's Report Form (TRF) At baseline assessment, children in both intervention and control groups will play a concentration game and be measured for EEG activity. The ADHD Rating Scale will be administered to the parents and teachers of all subjects in both study groups at weeks 4, 8, 12, 16, 20, 24, 28, and at 3 months post-intervention (week 32): The following will be completed at weeks 8, 20 and 32: 1. Child Behaviour Checklist (CBCL): parent 2. Teacher's Report Form (TRF): teacher 3. DISC-IV (week 8 and 20 only) 4. WIAT-III (at week 20 only) The subjects and their parents will make clinic visits for these scheduled assessments. All questionnaires to be completed by the teachers will be mailed to the child's school. They will then be returned to the Principal Investigator in a self-addressed envelop. Reminders will be sent at 14-day intervals if the teachers did not return the questionnaire in time. At weeks 0, 4, 8, 12, 16, 20, 24, 28 and 32, a clinician (who is blinded to the group assignment) will also complete the following: 1. Clinical Global Assessment Scale 2. Clinical Global Impression -Severity Scale 3. Clinical Global Impression – Improvement Scale The primary outcome will be change in the inattentive score on the ADHD Rating Scale as rated by teachers at week 8. Teachers' ratings are preferred as they are blind to the intervention received by the child. Secondary outcomes include inattentive score rated by parents on the ADHD Rating Scale at weeks 8, and 20, ADHD diagnosis status based on DISC, CBCL/TRF attention problem scores, clinician rated C-GAS and CGI scores and academic assessment. Subjects will be considered as 'drop-outs' if they: 1. Become lost to follow-up or discontinues intervention at any point. 2. Receive any medication (e.g. methylphenidate, atomoxetine, tricyclic anti-depressant) or supplement (e.g. omega-3 fatty acid) which can improve ADHD symptoms during the trial period. The parent/child will be asked about this each time they turn up at the clinic. POTENTIAL DIFFICULTIES Subjects may have difficulties making their way down to the clinic for the frequent interventions. As far as possible, we will attempt to avoid involving examination periods (May and October) during the 8-week intervention.

**F9 Please provide details on sample size and power calculation and the means by which data will be analyzed and interpreted (If applicable).**

Based on a moderate effect size of 0.4 on ADHD Rating Scale inattentive score improvement rated by teachers, power of 80% and type I error of 5%, a sample size of approximately 125 subjects per group is necessary. This includes an allowance for up to 25% losses to follow-up. Intervention groups will be compared for balance in baseline characteristics and in CONSORT flow chart. The primary outcome will be improvement on the ADHD Rating Scale scores at completion of the 20-session intervention. The main analysis will be based

on two-sample t-test. Continous Secondary outcomes will be analyzed by t-test or Mann-Whitney U test where appropprieate. Chi-square test or Fisher’s exact test will be applied for categorical secondary endpoints.

**F10 List all research related activities.**

Participants will be complete interviews, intervention and assessment in the clinic. Intervention:- BCI training, worksheetsAssessment:- Children: WIAT- Parents: C-DISC, ADHD Rating Scale, CBCL- Teachers: ADHD Rating Scale, TRF- Clinicians: CGI/CGAS

**F11 List all activities that are performed for routine diagnostic or standard medical treatment purposes.**

Subjects will be diagnosed for ADHD according to DSM-IV criteria by the psychiatrist before enrolment into this study.

**F12 Please describe the subject's visits (frequency and procedures involved). For studies with multiple visits, please attach study schedule. (If applicable)**

Please refer to the study schedule for subject's visits.

**BCI_Study Schedule_081209_ST.xls**

**F13 Discuss the potential difficulties and limitations of the proposed procedures and alternative approaches to achieve the aims.**

The pilot study revealed non-response rate among teachers to be as high as 40%. We will try to minimise responder burden on teachers by minimising the frequency and number of questionanires administered. We also intend to make the shorter questionnaire, ADHD Rating Scale available online to facilitate completion and timely submission, rather than using snail mail.

**F14 What are the Potential Risks to Subjects?**

From literature, the potential risks may include seizure or destabilization of the individual's mood state. To minimise such risk, individuals with seizure or severe psychiatric disorder will be excluded from participation.

**F15 What are the Potential Benefits to Subjects?**

All subjects will eventually receive Brain Computer Interface (BCI) intervention. These subjects may see improvement in their attention.

**F16 Preliminary Studies / Progress Reports. Please provide an account of the Principal Investigator's preliminary studies (if any) pertinent to this application.**

A pilot non-randomized trial to test the treatment protocol and its efficacy was conducted on 10 children with ADHD. Another 10 children with ADHD were recruited as controls. The 20–session intervention program was well accepted by the parents and children. The ADHD scales administered by parents and teachers both showed good test-retest reliability (>0.9). After 20 sessions of therapy, having adjusted for baseline score, children in the intervention group showed statistically significant difference in the attention problem score on the ADHD Rating Scale at 10 weeks compared to children in the control group, with a mean (SD) reduction of inattentive scores of 2.9 (4.0) for the treatment group vs. -0.5 (2.0) for control group $p<0.05$.

**F17 What is the estimated timeline for this study?**

**Estimated Start Date: 08-Mar-2010**

**Estimated End Date: 31-Dec-2012**

**F18 Does this study have a Study Protocol?**

○ Yes
◉ No

**F19 The PI is responsible for ensuring that all Study Subjects give informed consent before enrolling into the study.**

**Please select all the applicable consent scenarios.**

- ◉ Informed Consent will be taken for all study subjects.
- ○ Waiver of Informed Consent is requested for all study subjects.
- ○ A combination of both Informed Consent and Waiver of Consent is required for different study populations.

**G1 Describe the study protocol(s) to be used. Include information of the study drug / device / surgical procedures that will be used in the trial. If the study involves the use of study drug / device, describe how you plan to manage the receipt, handling, storage, utilization, and disposal of the study drug/device.**

Intervention: 3 sessions weekly for 8 weeks: - 1st session: 30-min BCI - 2nd session: 30-min BCI + 10-min worksheet

**G2 Please attach Investigator's Brochure, if applicable.**

**G3 Describe alternative treatments used at your institution for this condition.**

Stimulant/non-stimulant medicationPsychosocial treatment (Behavioural management)

**G4 Is this a placebo controlled trial?**

- ○ Yes
- ◉ No

**H1 How will potential subjects be identified? (Please tick all the applicable boxes)**

☑ Referral by attending healthcare professional
☑ Patients of study team
☐ Databases
☐ Other methods of subject identification

**H2 Who will make the first contact with subject (Enter NA if not applicable)?**

The treating doctor will inform parents of potential subjects. The contact number of these potential subjects will then be obtained.

**H3 How will the subject be contacted (Enter NA if not applicable)?**

The protocol administrator will contact parents of potential subjects to arrange a clinic visit for informed consent to be obtained.

**H4 Will any advertising / recruitment materials be used to recruit research subjects?**

○ Yes
◉ No

**H5 Will any other recruitment strategies be used? (Eg. Talks in public places, societies etc.)**

○ Yes
◉ No

**H6 What is the Recruitment Period (if applicable)? Please provide us with the approximate recruitment period.**

**Start Date: 31-Aug-2009**

**End Date: 31-Dec-2011**

**H7 Please indicate the length of time of the subject's direct involvement in the study. E.g. For clinical visits, examinations etc. (If applicable)**

The length of time will be 9 months.

**I1 Please state the target number of research subjects to be recruited for each study site in Singapore. If exact numbers are not available, please give an approximate number.**

**(Go back to Section B1 to add additional study site)**

| No. | Study Site | Recruitment Target Min | Recruitment Target Max | Males | Females | Children |
|---|---|---|---|---|---|---|
| 1 | Institute of Mental Health | 270 | 270 | 0 | 0 | 270 |

**I2 Is this study part of an international study?**

○ Yes
◉ No

**K1 Please list the inclusion criteria for research subjects in this study.**

1. Through clinical interview of their parents and teachers, as having satisfied the criteria as listed in the DSM-IV TR.2. Using the Diagnostic Interview Schedule for Children (DISC)

**K2 Please list the exclusion criteria for research subjects in this study.**

1. Present or history of medical treatment with stimulant medication and atomoxetine2. Co-morbid severe psychiatric condition or known sensorineural deficit e.g. complete blindness or deafness3. History of epileptic seizures4. Known mental retardation (i.e. IQ 70 and below)5. Predominantly hyperactive/impulsive subtype of ADHD

**K3 Please state the age group of the research subjects.**

**Lower Age limit 6 Lower Age option years**

**Upper Age limit 12 Upper Age option years**

**K4 Are there any recruitment restrictions based on the gender of the research subjects?**

◉ Yes

**Please give reasons for the gender restriction.**

ADHD affects males more than females; the approximate male/female ratio will be 9:1, as per that of the clinical population.

○ No

**K5 Are there any recruitment restrictions based on the race of the research subjects?**

○ Yes
◉ No

**K6 Do the potential research subjects have a dependent relationship with the study team (E.g. doctor-patient, employee-employer, head-subordinate, student-teacher, departmental staff relationship)?**

○ Yes
◉ No

**K7 Does the study involve any of the vulnerable research participants?**

◉ Yes

**Please select all the applicable categories.**

☐ **Pregnant Women, Foetuses and Neonates**
☑ **Children (persons who are less than 21 years of age)**
☐ **Prisoners**
☐ **Cognitively Impaired persons**
☐ **Others (E.g. mentally disabled persons, or economically or educationally disadvantaged persons.)**

○ No

**K8 Does the study involve any of the following?**

☐ inpatients
☑ outpatients
☐ healthy volunteers
☐ Not applicable

**M Research involving Children (Persons under the age of 21 years) - Please provide protocol specific information explaining how your proposed research project meets the following criteria.**

**M1 Describe whether appropriate studies have been conducted on animals and adults first and the data available from such studies is available to assess risks to children participating in the research.**

This study involves non-invasive intervention. Similar studies on brain-computer interface interventions have been done in children and adolescents with ADHD. We have also done a pilot trial.

**M2 Why does the research need to involve children? Can the research question be answered through alternative means?**

ADHD is a child psychiatric condition, with onset before the age of 7. As the symptoms may improve with age, and the phenotype of the illness may differ in adults, it will be inaccurate to extrapolate results in adults to children.

**M3 Describe how the relation of potential benefits to risks is at least as favourable to the participants as that presented by alternative approaches.**

This intervention is non-invasive, unlikely to cause any direct harm and may improve their ADHD symptoms. Standard treatment with medication can cause side effects which often cause parents concern.

**M4 Describe the additional safeguards that will be provided to protect rights and welfare of these vulnerable subjects.**

Subjects of both groups will receive behaviour management, a standard treatment for ADHD. All involved children will also be followed up by their child psychiatrist.

**M5 What are the provisions for obtaining the child's assent and parental permission? (Check all that apply)**

◉ Assent will be obtained from all children above 6 years old and Parental Permission will be obtained.

- ☑ Separate Assent Form (for ages 6 - 12)
- ☐ Provision for Signature of Child on Parental Consent Form (for ages 13-20).

**Please submit the appropriate document**

**BCI ADHD_assent form pilot_160410_ST.doc**

**BCI ADHD_assent form trial_160410_ST.doc**

○ Assent will not be obtained from the children. Only Parental Permission will be obtained.
○ Parental Permission will not be obtained from the parents. Only assent will be obtained.
○ Neither the children's Assent or Parental Permission will be obtained.

**P YES. Informed consent will be obtained from potential Research Participants before enrollment into the study.**

**The PI is responsible for ensuring that all Research Participants give informed consent before enrolling into the study. Please describe the consent process below.**

**P1 When will the consent process take place with the potential Research Participant?**

Informed consent from parents and assent from children will be taken during the first clinic visit (for baseline assessment).

**P2 Where will the consent process take place with the potential Research Participant?**

Consent will be taken in the clinic premises of the Child Guidance Clinic.

**P3 Who will conduct the consent process with the potential Research Participant?**

One of the study investigators/research associates will conduct the consent process.

**P4 Describe how the consent process described above (in consideration of the time and place where consent is taken, and the person taking consent) minimize the possibility of coercion or undue influence.**

Subjects will be given time to consider their participation in the study. They will return to the clinic for informed consent taking should they wish.

**P5 Do you anticipate a situation where obtaining informed consent from a potential Research Participant is not possible and informed consent will be taken from the legally acceptable representative (including spouse, parent, and guardian)?**

◉ No
○ Yes

**P6 Describe provisions to protect the privacy interests of Research Participants, where 'privacy interests' refer to interests of individuals to be left alone, free from intrusion and comfort with the proposed settings.**

The child should be accompanied by the parent during every visit to the clinic. All interventions will be performed in designated laboratory rooms within the clinic to ensure privacy.

**P7 Besides the Consent document, will any other materials or documents be used to explain the study to potential Research Participants? (eg. scripts, handouts, brochures, videos, logs, etc).**

◉ No
○ Yes

**P8 Will research participants receive any monetary payments (including transportation allowances) or gifts for their participation in the study?**

○ No
◉ Yes

**Please provide details of the gifts and payment (including the amount paid).**

$40 for each long visit (assessment) and $10 per short visit (intervention)

**P9 Will consent be documented in the form of a written and signed Research Participant Information Sheet and Consent Form?**

◉ Yes, all Research Participants will be given a copy of the Research Participant Information Sheet and Consent Form.

**Please attach a copy of the Information Sheet and Consent Form.**

○ No, Consent will not be documented. (E.g. verbal consent).

**P10 Consent Language**

**(i) Will the study enroll non English speaking subjects?**

○ No
◉ Yes

**a. If Yes, what are the possible languages that will be understood by the prospective participant or the legally acceptable representative?**
☑ Chinese
☐ Malay
☐ Tamil
☐ Others (state the language)

**b. Will the consent be communicated in a language that is understood by the prospective participant or the legally acceptable representative?**
○ No
◉ Yes

**c. How will the Non-English consent be documented?**
☑ Consent Document (Full) translated to the language understood by the prospective participant or the legally acceptable representative. - You may attach the translated consent document, if available. Otherwise, please submit the translated document after the English version has been approved by DSRB.

**- You may attach the translated consent document, if available. Otherwise, please submit the translated document after the English version has been approved by DSRB.**

☑ DSRB Short Form Consent Document Template - Please download the template from NHG Research Website. This document does not need to be reviewed and approved by DSRB if there are only administrative amendments to the Template.

**- Please download the template from NHG Research Website. This document does not need to be reviewed and approved by DSRB if there are only administrative amendments to the Template.**

☑ Other Short Form Consent Document - If you are not using the DSRB Short Form Consent Document Template, please submit the English version of the short form consent for review. You may also attach the translated consent document, if available. Otherwise, please submit the translated document after the English version has been approved by DSRB.

**- If you are not using the DSRB Short Form Consent Document Template, please submit the English version of the short form consent for review. You may also attach the translated consent document, if available. Otherwise, please submit the translated document after the English version has been approved by DSRB.**

**P11 Will the study be recruiting subjects under emergency situations, when prior consent of the subject is not possible, and the consent of the subject's legally acceptable representative, if present, should be requested?**
○ Yes
○ No

**P12 Do you have any additional comments regarding the Informed Consent process?**
◉ No
○ Yes

**R In general, to protect the Study Subject's confidentiality, research data should be coded, and the links between the Subject's identifiers and the codes should be stored separately from the research data.**

**R1 Coded / anonymous research data will be sent to the study sponsor, and therefore no research database will be created and stored in NHG?**

◉ No

**i. Describe where the research data will be stored. (ie: Network or stand-alone PC, and the physical location)**

All data will be stored on the principal investigator's office personal computer, which is password-protected, and located in a small office within the hospital which is locked when empty.

**ii. Who will have access to the research data, and how will access to the research data be controlled and monitored?**

The use of password protection on research study databases. All study participants are identified by unique identification numbers. Personal data (names, IC numbers, addresses and telephone numbers) will be kept in a separate file. Access to research study databases will be restricted and only study staff will be given computer passwords to access study data files with scheduled changes to passwords. All case report forms/data collection forms will be without identifiers and will be kept under lock and key.

**iii. Are there any other measures in place to protect the confidentiality of the research data?**

All patient identifiers will be delinked, and the subjects will be assigned a number instead.

**iv. Are there any research data sharing agreements with individuals or entities outside the Institution, to release and share research data collected?**

◉ No
○ Yes

**v. Describe what will happen to the research data when the study is completed.**

The database will be registered with the NHG standing database registry after the study is completed.

○ Yes

**R2 Will any part of the study procedures be recorded on audiotape, film/video, or other electronic medium?**

◉ No
○ Yes

**S1 Will any biological materials (such as blood or tissue) be used as part of the study? This includes both prospectively collected and existing biological materials.**

- ◉ No
- ○ Yes

**T The purpose of the Data and Safety Monitoring Plan is to ensure the safety and well being of Study Subjects, and the integrity of the data collected for the study. Depending on the type and risk level of the study, this may include the Principal Investigator, experts within the department or institution, independent consultants or a combination of the said persons.**

**T1 Who performs the data and safety monitoring? If there is a Data Safety Monitoring Board (DSMB), please submit the charter of the DSMB.**

The Prinicpal Investigator will ensure data safety and monitoring.

**T2 When and what safety data is monitored (Enter NA if not applicable)?**

All entered data will be monitored.

**T3 When and how is data integrity monitored?**

Data integrity will be ensured at all times during the study process. All hard copies of the data collection forms will not carry patient identifiers other than assigned subject numbers. One clinic room will be specially set aside for the intervention sessions, and will be lcoked when not in use.

**T4 What are the criteria for stopping the research?**

Patients are not expected to experience any problem when participating in these games. There is no invasive procedure involved. The research may however, be suspended if any part of the protocol is breached, or if there is any violation of standard research ethics. Since the patients will not be put on medication, the exit criteria to pull out of the study should their symptoms worsen is an increase in ADHD Rating Scale score of 20% of their baseline.

**T5 How will the outcome of data and safety monitoring be communicated to the study sites?**

There is only one study site.

**If any one of the study team member's curriculum vitae does not appear on this list, the CV must be uploaded through the user's profile.**

| No. | Study Site | Name | Study Role | CV |
|---|---|---|---|---|
| 1 | Institute of Mental H ealth | Guan Cuntai | Co-Investigator | |
| 2 | Institute of Mental H ealth | Lim Shih Hui | Co-Investigator | |
| 3 | Institute of Mental H ealth | Krishnan, Ranga R. | Co-Investigator | |
| 4 | Institute of Mental H ealth | Choon Guan Lim | PI | |
| 5 | Institute of Mental H ealth | Zhao Yudong | Co-Investigator | |
| 6 | Institute of Mental H ealth | Cheung Yin Bun | Co-Investigator | |
| 7 | Institute of Mental H ealth | Daniel Fung | Co-Investigator | |
| 8 | Institute of Mental H ealth | Lee Tih-Shih | Co-Investigator | |

**Your DSRB Application is now complete and ready for submission.**

Principal Investigator's Declaration

**I will not initiate this study until I have received approval notification from the DSRB and all applicable regulatory authorities.**

**I will not initiate any change in the study protocol without prior written approval from the DSRB, except when it is necessary to reduce or eliminate any immediate risks to the Research Participants. Thereafter, I will submit the proposed amendment to the DSRB and all applicable regulatory authorities for approval.**

**I will promptly report any unexpected or serious adverse events, unanticipated problems or incidents that may occur in the course of this study.**

**I will maintain all relevant documents and recognise that the DSRB staff and applicable regulatory authorities may inspect these records.**

**I understand that failure to comply with all applicable regulations, institutional and DSRB policies and requirements may result in the suspension or termination of this study.**

**I declare that there are no existing or potential conflicts of interest for any of the investigators participating in this study.**

**By checking the "I agree" box, you confirm that you have read, understood and accept the Principal Investigator's Declaration**

☑ **I have read and agree to the above declaration.**

**Principal Investigator: Lim Choon Guan**
